# The combined effect of decreased stomatal density and aperture increases water use efficiency in maize

**DOI:** 10.1038/s41598-025-94833-1

**Published:** 2025-04-21

**Authors:** Larissa Barl, Betina Debastiani Benato, Nikita Genze, Dominik G. Grimm, Michael Gigl, Corinna Dawid, Chris-Carolin Schön, Viktoriya Avramova

**Affiliations:** 1 Plant Breeding, TUM School of Life Sciences, Technical University of Munich, 85354 Freising, Germany; 2 Bioinformatics, TUM Campus Straubing for Biotechnology and Sustainability, Technical University of Munich, 94315 Straubing, Germany; 3 Bioinformatics, Weihenstephan-Triesdorf University of Applied Sciences, 94315 Straubing, Germany; 4 Functional Phytometabolomics, TUM School of Life Sciences, Technical University of Munich, 85354 Freising, Germany

**Keywords:** Plant physiology, Machine learning, Image processing, Heat, Transgenic plants

## Abstract

Stomata play a crucial role in balancing carbon dioxide uptake and water vapor loss, thereby regulating plant water use efficiency (WUE). Enhancing WUE is important for sustainable agriculture and food security, particularly for crops such as maize (*Zea mays* L.), as climate change and growing global food demand exacerbate limitations on water availability. Genetic factors controlling stomatal density and levels of the plant hormone abscisic acid (ABA) in leaves, which affect stomatal aperture, are key determinants of stomatal conductance (g_s_) and intrinsic WUE (iWUE). In this study, we demonstrate that stomatal density and stomatal aperture have a combined effect on g_s_ and iWUE in maize. Using near-isogenic lines (NILs) and CRISPR/Cas9 mutants, we show that combining reduced stomatal density and reduced stomatal aperture can improve iWUE without compromising photosynthesis. This effect is pronounced at both, optimal and high temperatures. These findings highlight the potential of targeting multiple stomatal traits through genetic stacking to enhance WUE, offering a promising strategy for crop adaptation to water-limited environments.

## Introduction

Improving plant water use efficiency (WUE) is crucial for sustainable crop cultivation in times of climate change with limited water availability for agriculture^1,2^. WUE is the ratio of crop productivity (grain or biomass yield) per unit of water consumed^3^. With improved WUE, farmers can reach higher productivity of crops even when water resources are limited, which is especially important in arid or semi-arid areas or in regions, where crops frequently face chronic stress. To achieve global food security, plant production must capitalize on the existing arable land, thus crops with high WUE are crucial. Genetic improvement of WUE has impact on water management and reduces the risk of crop failure. While optimising agricultural practices to achieve better crop and water management could provide immediate improvement of WUE during the field season, breeding for crop varieties with superior WUE is a sustainable, long-term solution for global food security^4^.

WUE can be assessed at different scales (leaf, plant or canopy)^5^. WUE at the plant level, WUE_plant_, is highly correlated with intrinsic WUE (iWUE), measured at the leaf level and calculated as the amount of CO_2_ assimilated (A) relative to stomatal conductance (g_s_)^6^. Stomatal conductance estimates the rate of gas-exchange (CO_2_ uptake and water loss through water vapour) through the stomata and is a function of stomatal properties such as aperture, density and size. Genetic alterations in each of these anatomical characteristics have been shown to affect iWUE^7–10^.

Stomatal aperture depends on the turgor of the guard cells, which is controlled by environmental factors such as concentration of atmospheric CO_2_ and light intensity, and levels of the plant hormone abscisic acid (ABA) as an endogenous factor^11^. High levels of ABA initiate stomatal closure, which causes decreased g_s_. This results in water conservation, but in most cases a concomitant reduction in photosynthesis has to be accepted. However, accumulation of ABA in the leaves, due to defective ABA catabolism in maize^8^ or increased sensitivity of guard cells to ABA in Arabidopsis and wheat^7,12^, have led to a significant reduction in g_s_ with little or no effect on A, thereby increasing iWUE and WUE_plant_.

While stomatal aperture provides a fast response to a changing environment, alterations in stomatal density (SD) and size ensure long-term adjustments of g_s_. These two anatomical properties usually have an inverse relationship, due to geometric constraints of the leaf, but also due to their functionality^13^. High densities of smaller stomata are usually more responsive, leading to faster adaptation in stomatal conductance and photosynthesis under fast changing environmental conditions^14,15^. In stable environments, reduced density has been shown to positively affect iWUE^9,16–18^. Similar to aperture, stomatal density is modulated by environmental factors such as air humidity, temperature, CO_2_ concentration and light intensity^19^. It also depends on genetic factors involved in epidermal patterning, cell fate or hormonal signalling^19^. At the molecular level, stomatal differentiation is regulated by mechanisms involving the transcription factor SPEECHLESS (SPCH)^21–23^. It plays a central role in stomatal formation, initiating it in meristematic cells and its overexpression leads to higher SD in Arabidopsis^22,23^. Another important player in stomatal formation is ABA. In Arabidopsis, it has been demonstrated that ABA controls not only stomatal aperture but that it is also involved in stomatal development^26–28^. Arabidopsis mutants defective for members of the gene family encoding ABA 8′-hydroxylases, key enzymes of the catabolic pathway of ABA^29^, have been shown to accumulate ABA and to have lower SD^26^.

Altering the gene network controlling stomatal development is the main tool reported in the literature to reduce SD^30^. The most commonly used targets additional to transcription factors are members of the epidermal patterning factor (EPF) signalling molecules^31–36^, the receptor-like kinases ERECTA^37,38^ and stomatal density and distribution1 (SDD1) subtilisin-like serine protease^39,40^. The targeted reduction in SD is sometimes compensated by enlarged stomatal size^31,38^ or increased stomatal aperture^17,18,41^, which mitigates the effect on g_s_ and iWUE^39,41^. However, smaller stomata were also observed in plants with low SD^17,42^ and in many cases WUE was improved^33,36,38^. Most of these studies were conducted in eudicotyledon (dicots) and translating these findings to monocotyledons (monocots) is limited by differences in epidermal cell patterning and stomatal morphology between dicots and monocots. While in dicots the formation of stomata is scattered over the epidermis of the mature leaf, in monocots it occurs along a spatiotemporal gradient and the cell fate is decided at the basal leaf meristem, after which cells move upwards as the leaf expands^20^. Therefore, in monocots SD is already determined in the meristem during early leaf development and does not change as the leaf grows. Thus, many examples in the literature demonstrate the improvement of iWUE through the reduction of g_s_ either mediated by lower stomatal density^33,36,38^ or by reduced aperture caused by elevated ABA levels in leaves^8^. However, because stomatal characteristics are often interlinked and functionally coordinated and the same factors such as ABA regulate both stomatal aperture and development, it is difficult to separate the two traits genetically. Therefore, studies comparing their individual and joint effects on g_s_ and iWUE and estimating their importance in different environmental conditions are limited.

Maize is a suitable model to address this knowledge gap as it is a C_4_ monocot plant. Due to their CO_2_ concentrating mechanism, C_4_ species are less sensitive to variations in intracellular CO_2_ compared to C_3_ species^43^ and therefore experience less stomatal limitations of A^44^. This makes maize ideal for studying the effects of stomatal variation without confounding photosynthetic limitations. In addition, the developmental gradient of the leaf, which maize exhibits as a monocot, enables the separation of stomatal formation, occurring in the meristematic zone, and stomatal closure, which happens in the mature zone of the leaf^21^. This spatial separation allows the isolation of genetic factors exerting distinctive control on either of the two processes.

Here, we aimed to compare the individual effects of the two stomatal properties on iWUE in maize and hypothesize that their combined effect leads to greater improvement in WUE.

**Fig. 1:**
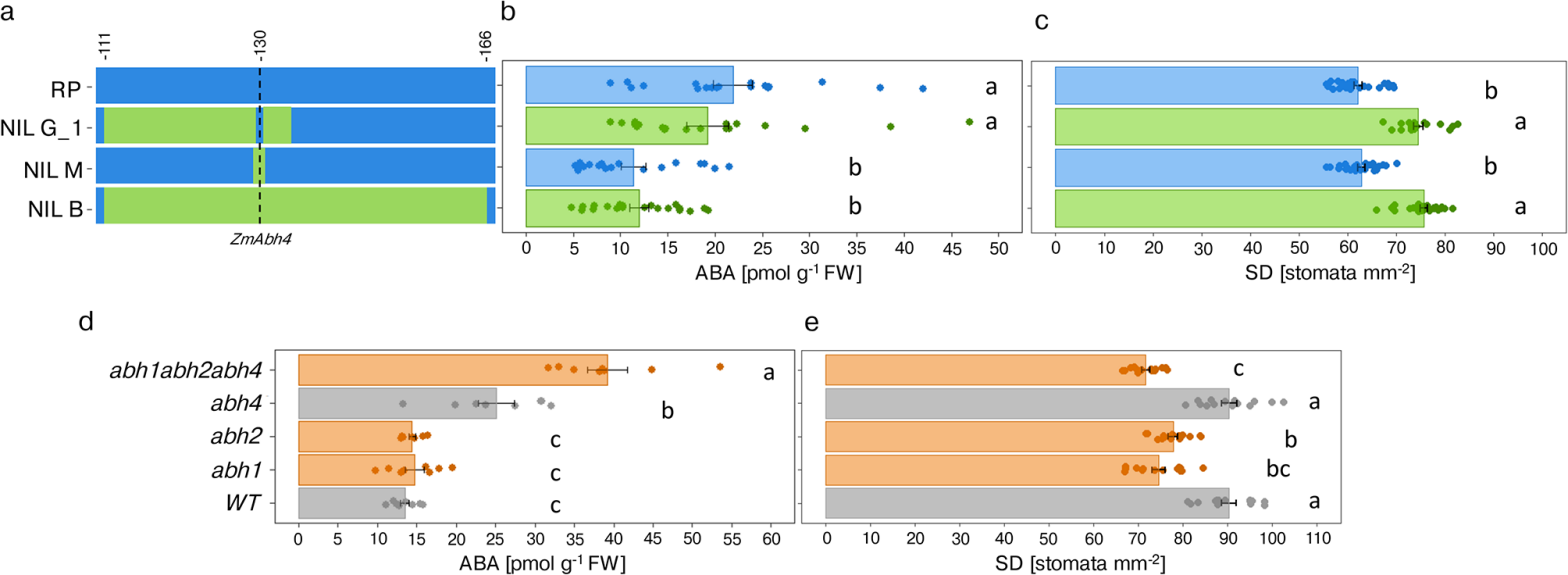
Abscisic acid (ABA) leaf concentrations and stomatal density (SD) in near isogenic lines (NILs) and CRISPR/Cas9 *ZmAbh *mutants. (**a**) Introgressions from a donor parent (dark green) into the genomic background of recurrent parent (RP; dark blue) in the region between 111 and 166 Mb on chromosome 7 carried by near isogenic lines NIL B, NIL M, and NIL G_1. Recombination breakpoints were determined with the 600 k Axiom™ Maize Genotyping Array (Supplementary Table S2). The position of the *ZmAbh4* gene is indicated by a dotted line. (**b**) ABA content (pmol g⁻^1^ fresh weight (FW)) measured in fully developed leaves of NILs (n = 20–21 plants). (**c**) Stomatal density (SD) of NILs determined by deep learning-based stomata detector (n = 19–26 plants). Light blue and green bars indicate genotypes with low or high stomatal density, respectively. (**d**) ABA content in wild-type (WT) and mutants (*abh1, abh2, abh4*, and *abh1abh2abh4*) measured in fully developed leaves (n = 8 plants). Orange and grey bars indicate genotypes with low or high stomatal density, respectively. (**e**) SD of mutants determined by deep learning-based stomata detector (n = 14 plants). Bar charts show means ± SE. Different letters indicate significant differences in pairwise comparisons with Benjamini–Hochberg correction (P < 0.05).

In a previous study, we identified the near isogenic maize line NIL B, that shares 97.25% of its genome with its recurrent parent (RP). The two lines differ by a 55 Mb introgression on chromosome 7 that affects leaf ABA levels, stomatal density and WUE (Fig. 1A)^45^. In a forward genetic approach, we identified the gene *ZmAbh4*, encoding an ABA 8′-hydroxylase, as causative for the effect on leaf ABA levels^8^. RP carries a defective allele leading to disrupted ABA catabolism and elevated ABA levels in leaves compared to NIL B^8,45^.

Here, we demonstrate that stomatal density and leaf ABA levels are controlled by independent genetic factors linked in the 55 Mb introgression and that these two traits have an additive effect on g_s_ and iWUE, which is pronounced both under optimal and high temperatures. We show this effect in two different genomic backgrounds. Using single and triple CRISPR/Cas9 mutants, we demonstrate that the genetic stacking of genes controlling different stomatal properties could be a suitable approach to achieve significant improvement of WUE, especially under high temperatures.

## Results

### Stomatal density is independent of leaf ABA levels in NILs and *ZmAbh* mutants

To study the individual and combined effects of stomatal aperture and stomatal density on WUE, we analysed three maize near-isogenic lines, NIL B, NIL G_1 and NIL M, carrying different fragments of a genomic region on chromosome 7, associated with these traits (Fig. 1A), and the RP. Consistent with previous studies^8,45^, NIL B, carrying the entire 55 Mb introgression, showed lower leaf ABA concentrations and higher stomatal density.

We first addressed the question whether the effects on ABA leaf levels and stomatal density are due to a pleiotropic action of *ZmAbh4* or rather due to two separate genetic factors linked in the genomic fragment carried by NIL B. In Arabidopsis, mutants defective for genes encoding ABA 8'-hydroxylases have likewise shown ABA accumulation in leaves and, in addition, reduced stomatal density^26^. However, NIL M, carrying a small introgression (200 kb) with the functional *ZmAbh4* allele (Fig. 1A), and lower ABA leaf levels, showed no significant change in stomatal density relative to RP (Fig. 1B, C). In contrast, NIL G_1, with two introgressions covering 23 Mb of the 55 Mb genomic introgression in NIL B, but lacking the functional *ZmAbh4* allele (Fig. 1A), showed significantly higher stomatal density but no difference in ABA levels (Fig. 1B, C). These findings demonstrate that genes located in the 23 Mb genomic region, in which NIL G_1 and NIL M differ, control stomatal density independently of the *ZmAbh4* allele and leaf ABA levels.

To further investigate the mechanisms of the genetic control of stomatal density in the 23 Mb region, we examined the expression of the transcription factor *ZmSpch* in the leaf meristems of the four NILs. This transcription factor is essential for stomatal differentiation and exhibited significantly higher transcript levels in lines with increased stomatal density (NIL B and NIL G_1) compared to those with lower density (NIL M and RP; Supplementary Fig. S1), consistent with previous studies in maize^21^. These results suggest that genetic factors located within this segment regulate a key transcription factor for SD. Guard cell formation was not affected by the introgression (Supplementary Fig. S2). To facilitate phenotyping of SD, we developed a deep learning-based stomata detection tool (see Materials and Methods for a detailed description).

The *ZmAbh* family includes five homologs in maize, of which *ZmAbh1*, *ZmAbh2* and *ZmaAbh4* are expressed in leaves^46,47^. We generated CRISPR/Cas9 null mutants for each of the three genes in the B104 genomic background as well as a triple mutant combining three independent null mutations for the same genes (Supplementary Fig. S3). Similar to the results from the NILs (Fig. 1 A, B), the *abh4* mutant showed significantly higher ABA leaf content compared to the wild type (WT; B104), but no effect on stomatal density (Fig. 1D, E). The *abh1* and *abh2* mutant lines, on the other hand, demonstrated no significant effect on leaf ABA levels, but significantly decreased stomatal density compared to WT. In the triple mutant, a cumulative effect was observed as the leaf ABA levels were increased by threefold compared to WT and stomatal density was significantly decreased (Fig. 1D, E). These results indicate that i) similar to what has been observed in Arabidopsis^26^, mutants for genes encoding ABA 8′-hydroxylases (*abh1* and *abh2*) have decreased stomatal density, however *ZmAbh4* does not influence this trait; ii) different members of the *ZmAbh* family regulate stomatal aperture through leaf ABA accumulation (*ZmAbh4*) or stomatal density through a not yet characterized but probably spatially restricted effect (*ZmAbh1* and *ZmAbh2*) in maize, and both traits are affected in the triple mutant (*abh1abh2abh4*). Thus, the two sets of independent genetic material analysed in this study, NILs and CRISPR/Cas9 mutants, offer the unique opportunity to genetically separate the two stomatal traits, stomatal aperture and stomatal density, and to study their individual and combined contribution to stomatal conductance and water use efficiency.

### Lower stomatal density and stomatal aperture improve iWUE

To understand the effect of variation in SD and stomatal aperture on water saving and photosynthesis, we performed gas-exchange measurements on the NILs and the *ZmAbh* CRISPR/Cas9 mutants in two temperature regimes, 28 °C as control and 35 °C for high temperature treatment.

**Fig. 2:**
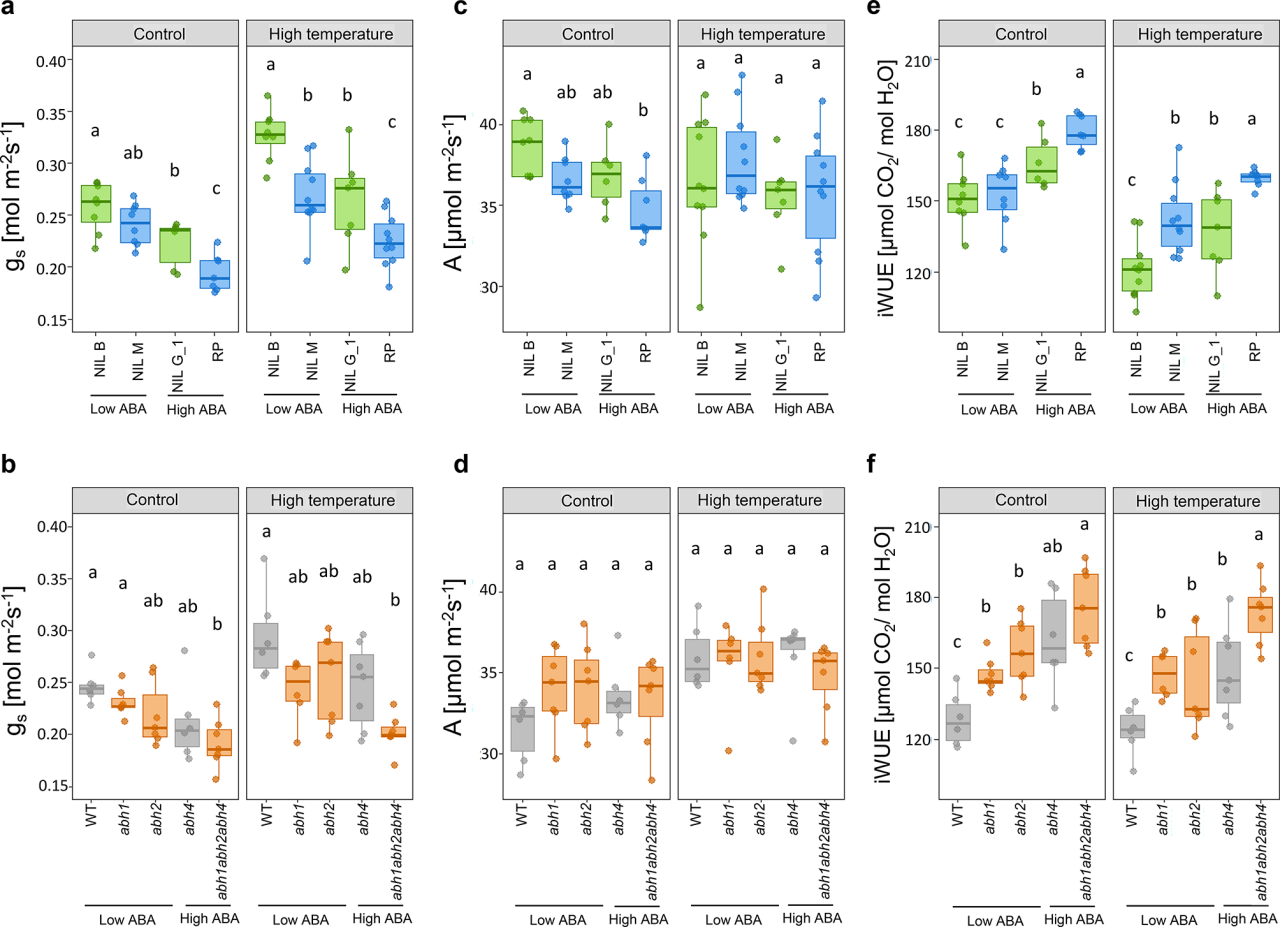
Effects of stomatal density (SD) and abscisic acid (ABA) levels on gas-exchange under control and high temperature treatment. Stomatal conductance (g_s_) under control and high temperature treatment for (**a**) NILs (n = 9–11 plants for control and n = 10–13 plants for high temperature treatment) and (**b**) *ZmAbh* mutants and their wild type (WT; n = 6–7 plants for both treatments). For the NILs, light blue and green bars indicate genotypes with low or high stomatal density, respectively. For the WT and mutant lines, orange and grey bars indicate genotypes with low or high stomatal density, respectively. CO_2_ assimilation rate (A) measured in the same conditions for NILs (**c**) and mutants (**d**). Intrinsic water use efficiency (iWUE) calculated from g_s_ and A for NILs (**e**) and mutants (**f**) across conditions. Boxplots represent the median (center line), upper and lower quartiles (box limits), and 1.5 × interquartile range (whiskers). Different letters indicate significant differences in pairwise comparisons within conditions with Benjamini–Hochberg correction (P < 0.05). A two-way ANOVA was performed to assess the effects of genotype, treatment and their interaction for each trait (g_s_, A and iWUE). ANOVA derived P-values are listed in Supplementary Table S1.

The high temperature treatment caused significantly higher g_s_ values across all NILs compared to the control (P < 0.001, Fig. 2A, Supplementary Table S1). Under control conditions, RP with low SD and high ABA leaf levels, which reduce stomatal aperture, exhibited the lowest g_s_ among the NILs (Fig. 2A). NIL G_1 has a higher SD than RP with similar leaf ABA levels and exhibited significantly increased g_s_ compared to RP. Similar to RP, the SD of NIL M is low, but its ABA levels are lower than in RP, allowing increased stomatal aperture. This also resulted in an increase in g_s_ of NIL M compared to RP (Fig. 2A). NIL B, combining high SD and increased stomatal aperture, displayed the highest g_s_ among all lines (Fig. 2A). This combined effect of SD and stomatal aperture on g_s_ observed under control at 28 °C was similar at 35 °C.

Similar results were obtained with the mutant lines (Fig. 2B). As with the NILs, g_s_ values generally increased with higher temperature (P < 0.001, Supplementary Table S1). In both conditions, the WT line B104, with high SD and low ABA levels, had the highest g_s_ (Fig. 2B). The *abh1* and *abh2* mutants with lower stomatal density compared to the WT, yet similar leaf ABA levels showed a strong trend towards reduced g_s_. The g_s_ of the *abh4* mutant, with the same SD as WT but higher ABA levels, was also decreased. In the *abh1abh2abh4* triple mutant, where low SD was combined with high ABA levels, the lowest g_s_ was observed in both conditions (Fig. 2B).

Overall CO_2_ assimilation of the NILs did not differ between control and high temperature treatments (Fig. 2C), however it was significantly elevated in WT and mutants in higher temperatures compared to the control conditions (P < 0.001, Fig. 2D, Supplementary Table S1). Under control conditions, NIL B assimilated significantly more CO_2_ than RP, but this observed advantage disappeared at 35 °C, where all NILs had similar values for A (Fig. 2C). The mutants and the WT showed no differences in CO_2_ assimilation in either treatment (Fig. 2D).

The higher temperature caused a significant decrease in iWUE for all the NILs compared to control conditions (P < 0.001, Fig. 2E, Supplementary Table S1), with the most pronounced difference in NIL B. This decrease in iWUE was not observed in the WT and the mutants (Fig. 2F), most likely due to the moderate increase in CO_2_ assimilation at high temperature. Under both conditions, RP and the triple mutant *abh1abh2abh4* displayed the highest water use efficiency among the respective set of genotypes (Fig. 2E, F). Under control conditions, changes in stomatal aperture contributed to a larger extent to differences in iWUE than SD. NIL G_1 had significantly higher iWUE compared to NIL B and NIL M. In the mutants, the differences in iWUE between *abh1*, *abh2* and *abh4* were not significant, but the same trend was maintained. Under high temperature conditions, on the other hand, the contribution of SD to the observed changes in iWUE became more pronounced, especially in the NILs (Fig. 2E), where iWUE of NIL M and NIL G_1 were no longer significantly different.

Plant growth measurements of leaf elongation rate (LER) and plant height at developmental stage V4 showed no significant differences among the NILs, thus demonstrating that no shoot growth penalties accompanied the improvement of iWUE (Supplementary Fig. S4). For the mutants, similar observations were made. The triple mutant showed slightly lower LER compared to the WT and the single mutants, however this was not reflected in its plant height (Supplementary Fig. S4).

These findings show that combining low SD with high ABA levels in leaves enhances iWUE. This effect was confirmed in two independent plant genetic materials under control and under high temperature conditions. Additionally, our results highlight that lowering SD alone significantly improves iWUE at higher temperatures.

## Discussion

WUE is an important crop performance parameter and its improvement allows crops to achieve higher yields with less water, which is crucial in the face of water scarcity and climate change. Generating crops with improved WUE can alleviate pressure on fresh water resources, reduce irrigation costs, increase the resilience of crops to unpredictable drought conditions, and help maintain agricultural productivity in water-limited regions^48^. Additionally, improving WUE contributes to sustainable agricultural practices by minimizing water waste^49^.

The relationship between stomatal density, stomatal aperture and stomatal conductance has been extensively studied, highlighting the potential of altering these traits to enhance WUE, but it was uncertain how comparable the individual effects of reduced stomatal density and stomatal aperture are in their contribution to regulating iWUE in different environmental conditions. A meta-analysis by Lunn et al.^41^ found that reductions in SD were generally correlated with lower g_s_ and improved WUE across both monocots and dicots. This correlation supports findings where targeted genetic modifications reduced SD and enhanced WUE with minimal impact on photosynthesis^17,18,50^. These studies demonstrate that changes in SD directly affect g_s_, which is in line with our findings. In this study, we show that g_s_ can be reduced by the effect of elevated leaf ABA levels leading to reduced stomatal aperture. The two sets of independent maize material used in this study allowed us to investigate each of the two traits, SD and stomatal aperture, separately in two different genetic backgrounds and examine their combined effects on iWUE. Our results show, that reduced SD and reduced stomatal aperture have a combined effect on the increase in iWUE, which is greater than altering either trait individually in both control and high temperature conditions. Additionally, this combination of traits leads to little or no effect on plant growth in the null mutants (Supplementary Fig. S4). For the NILs, as shown in our previous and current research, this combination does not result in trade-offs regarding key agronomic factors such as germination, plant growth, or yield (Supplementary Fig. S4)^8^. Translating the increased WUE from controlled to field conditions could be challenging due to the effect of environmental variables such as temperature, water availability, and nutrient supply which are difficult to control in field conditions^51^. However, we have also demonstrated that the WUE of RP is significantly improved compared to NIL B in field trials and stable over several years with no observable trade-offs in agronomic traits^45,51^.

High temperatures significantly increase stomatal conductance in both monocots and dicots to enhance evaporative cooling^52,53^. This response often leads to rapid water depletion during prolonged heat events^52^. Maize grows optimally at temperatures below 33 °C, and above this temperature it is subjected to mild heat stress, showing symptoms such as altered metabolic activity, changes in stomatal conductance and increased production of heat shock proteins^54^. Our results show that while under optimal conditions the effect of ABA leaf levels controlling stomatal aperture has a greater contribution to changes in iWUE than SD, the effect of SD becomes more prominent under higher temperature (Fig. 2). At higher temperatures, temperature signalling pathways primarily regulate stomatal behaviour and ABA plays only a secondary role by shifting the threshold for stomatal opening^55,56^. When plants maximize stomatal aperture to enhance cooling, SD determines the extent of water loss. A reduced SD could therefore provide an adaptive advantage by balancing transpirational cooling with water conservation. Thus, plants with lower SD may mitigate excessive water loss when the temperature is higher, making the role of SD in g_s_ and WUE regulation more evident.

Stomatal density is a polygenic trait influenced by a wide array of genetic and environmental factors. While key transcription factors such as SPCH, are essential for stomatal development, their regulation can be modulated by both ABA-dependent^57^ and ABA-independent pathways^58,59^. The 23 Mb introgression shared by NIL B and NIL G_1 does not include a sequence encoding SPCH, suggesting that the transcription factor is regulated indirectly by other genetic elements within the introgression to result in increased SD. This indicates that one or even multiple genes in the introgression could be influencing SD, and further fine-mapping and functional analyses are required to identify them. The mechanisms altering SD observed in the *abh1* and *abh2* mutants are independent of those in the NILs since the mutations in the defined genes are the only genetic differences compared to the wild type line. We show that *ZmAbh1* and *ZmAbh2* are regulators of stomatal density in maize. Their precise roles and the underlying molecular processes still remain to be characterized. Investigating maize native genetic diversity and allelic variations of *ZmAbh1* and *ZmAbh2* could provide deeper insights into their roles in improving water use efficiency for breeding programs.

Stomatal density is laborious to assess when stomata are counted manually in the leaf epidermis. We used stomatal density data generated in this study to apply a deep learning-based method for automating this step and thus facilitating high throughput phenotyping. We detected and quantified stomatal density in maize leaves using nail polish imprints. Nail polish imprints have been established as part of a routine method for analysis of stomatal traits in dicots^60–62^, because they provide a practical solution for high throughput phenotyping in the field. The computational detection tools developed for automated stomata phenotyping on these imprints are not suitable for monocots such as maize due to the differences in stomatal complex morphology^20^. Monocot-specific phenotyping platforms for stomatal properties^63–65^ rely on more complex sample preparation techniques or specialized equipment, that are not suited for remote field applications. Our tool fills this gap by offering a low-cost, easily scalable solution based on nail polish imprints, making it ideal for high throughput and/or resource-limited research. While our focus has been on optimizing the model to predict stomatal density, we will now extend its application to add the detection of additional stomatal traits.

The complex regulation of SD offers numerous potential targets for improving WUE in crops. By understanding the genetic networks that control stomatal development, we can explore strategies to fine-tune SD and enhance WUE without compromising yield both by targeting ABA-dependent and independent pathways to modulate SD in combination with genes that directly regulate ABA levels in leaves, such as *ZmAbh4*. Such genetic stacking has been demonstrated as a promising strategy to improve complex quantitative traits in crops such as drought^66^ and salinity^67^ tolerance and even combined resistance to different stress factors^68,69^. The additive effect of stomatal density and stomatal aperture on iWUE shown in this study, offers the opportunity for stacking different genetic factors controlling water use efficiency by conventional or genetic engineering approaches. Potential synergetic effects of stomatal properties with other traits contributing to WUE improvement such as root morphology and architecture and cuticle development will be explored further. Numerous genetic factors contributing to these traits have been identified^48,70^, some of which even control root and stomatal traits pleiotropically^71^. Therefore, discovering targets for genetic stacking with further additive effects on WUE holds great potential to contribute to sustainable crop cultivation in times of climate change.

## Material and methods

### Plant material

Two maize (*Zea mays* L.) lines, RP and NIL B, previously described by Avramova et al., 2019^45^, together with their progenies, NIL G_1 and NIL M, were used in this study. The genomic background of these lines was tracked and their recombination breakpoints and introgression lengths were determined by genotyping with the 600 k Axiom™ Maize Genotyping Array^72^ (Supplementary Table S2).

Additionally, CRISPR/Cas9 mutants of the *ZmAbh* gene family in the B104 genomic background were investigated. The *abh1* and *abh4* mutants were generated as previously described^8^. The *abh4* mutant used in this study corresponds to the *abh4.41* mutant from Blankenagel et al. 2022^8^. The *abh1* mutant originated from an independent mutation event and corresponds to *abh1.27* in Supplementary Fig. S3. The CRISPR/Cas9 mutant of the *ZmAbh2* gene, which corresponds to *abh2.21*, was generated, employing two gRNAs (GATGGCGCTCAGCCGCTTGC and AACTCACGATGACCGCCTTG). The triple mutant *abh1abh2abh4* was generated by crossing three different independent mutants of *ZmAbh1*, *ZmAbh2* and *ZmAbh4*. All CRISPR/Cas9-mediated mutations were predicted to result in non-fuctional proteins based on *in silico* analyses . For all alleles, except for the single *abh1* mutant, 50%-80% of the protein is missing, implying lack of function (Supplementary Fig. S3). For the single *abh1* mutant, nearly 20% of the sequence is missing, including a highly conserved region among ABA 8'-hydroxylases across species and therefore most probably essential for the protein function (Supplementary Fig. S5). In all mutants, presence of the wild type alleles of the non-mutated *ZmAbh* genes was verified by sequencing.

### Growth conditions

Plants were cultivated in a growth chamber at 28 °C/ 25 °C day/night, RH 65%, 16 h/8 h light/night, 800 μE m^−2^ s^−1^ photosynthetically active radiation (PAR) until V5 and half of the plants were transferred to another chamber at 35 °C/ 25 °C day/night, RH 65%, 16 h/8 h light/night one week before the measurements were done. For all experiments, plants were arranged in a randomized complete block design. The developmental stages of maize were determined according to https://www.pioneer.com/us/agronomy/staging_corn_growth.html.

### Plant growth measurements

Leaf length was measured for three consecutive days with a ruler on the emerging seventh leaf from the moment it was visible among the older leaves. Leaf elongation rate (LER) was calculated as the difference in length divided by the time difference between successive measurements. Plant height was measured with a ruler at developmental stage V4 for all genotypes.

### ABA quantification

ABA and its metabolites were quantified on fully developed leaf 6 for the NILs and fully developed leaf 7 for *ZmAbh* mutants as described by Blankenagel et al., 2022^8^.

### Transcript level measurements

To harvest the meristematic part of leaf 5 while it is developing, five individual plants per genotype in vegetative stage V3 were cut as close as possible to the roots and all leaves up to the leaf of interest, as well as small leaves enclosed by the leaf of interest, were removed. RNA was extracted from the 3 cm segment starting from the base of the leaves using a modified guanidine hydrochloride protocol^73^, followed by DNase digestion and first-strand cDNA synthesis (Maxima H Minus Kit, random hexamer primers, K1652, Thermo Scientific, MA, USA). Reverse-transcription quantitative PCR (RT-qPCR) to measure transcript levels was conducted on all five replicates per genotype, each with three qPCR reactions, using KAPA SYBR FAST (Thermo Fisher Scientific, MA, USA) in a QuantStudio™ 3 system (Thermo Fisher Scientific, MA, USA). The primer sequences used for the gene *ZmSpch* were taken from Xiang et al., 2021^50^. The transcript quantities of each replicate were calculated relative to the housekeeping gene *ZmMep* (primers: 5′-TGTACTCGGCAATGCTCTTG-3′, 5′- TTTGATGCTCCAGGCTTACC-3′).

### Gas exchange measurements

Gas exchange parameters were measured with an LI-6800 portable photosynthesis system (LI-COR Inc., Lincoln, NE, USA) on the youngest fully expanded leaf at plant stage V6. Measurements were performed at a CO_2_ concentration of 400 ppm, flow rate of 500 μmol mol^−1^, photosynthetic photon flux density of 1,500 µmol m^−2^ s^−1^, RH of 65% and leaf temperature of 28 °C for control plants or 35 °C for plants in the high temperature treatment. The measurements were recorded every 30 s for 20–45 min. The values for A and g_s_ in the stable phase of the recording of 10 measurements were used to calculate the respective mean values. Intrinsic WUE was calculated as A/g_s_.

### Computer vision-based characterization of stomatal density

Stomatal density was assessed on leaf 6 of plants at developmental stage V6. Leaf imprints from the abaxial side of the leaf were made using nail polish as described by Avramova et al., 2019^45^, with minor modifications. Imprints taken from both regions flanking the main vein were transferred to a microscope slide using a transparent cellophane tape, and nine pictures were taken with an OLYMPUS BX61 microscope (OLYMPUS, Tokyo, Japan), covering different regions of the imprints. The pictures were taken at a magnification of 10 × and a resolution of 2448 × 1920 pixels. The generated pictures are publicly available on Mendeley data: https://www.doi.org/10.17632/2bfcpc6bpj.1. The number of stomata was predicted by a deep learning-based computer vision method (see below) and by manually counting them for validation. Density was calculated as the average of predicted counts of nine pictures divided by the picture area.

### Data pre-processing

To develop our stomata detection model, we selected a diverse dataset of 250 grayscale microscope images of maize leaves to ensure high variability. Each image was manually annotated with bounding boxes, resulting in over 10,000 labelled stomata.

### Object detection framework

Object detection is a computer vision task that involves identifying and localizing objects within an image or video. It is a well-established field of machine learning, with models ranging from one-stage object detectors (Single-Shot-Detectors^74^ or the You-Only-Look-Once^75,76^ family) to two-stage object detectors (R-CNN^77,78^ family). For our study, we chose Faster R-CNN for its superior accuracy, as real-time predictions were not required for our use case. Additionally, we used transfer learning^79^ to benefit from pre-trained image-based features on large-scale datasets (ImageNet^80^ in our case). Specifically, similar to Genze et al. 2020^81^, we fine-tuned a Faster R-CNN-based object detector with a ResNet50 backbone to detect multiple stomata in microscopy images. ResNet50 is a deep residual neural network, consisting of 50 layers. So-called skip connections are used between layers to help overcome vanishing and exploding gradients and combat overfitting^82^. Faster R-CNN is an anchor-based model that consists of two sub-networks. The first one is a region proposal network (RPN) and is used to generate proposals of regions of interest. Afterwards, a feature vector is extracted by the pre-trained ResNet50 backbone to discriminate if the proposed region is a stomata or is part of the background. Both sub-networks are trained end-to-end by minimizing a cross-entropy loss^83^ for classification and a L1-loss for bounding box regression, computed separately for the RPN and backbone.

### Model training and hyperparameter optimization

Our dataset contained images with various imperfections that originated from the image acquisition process. These included out-of-focus areas caused by variations in imprint thickness and air bubbles introduced during the imprinting procedure. To address this, we divided the images into three groups: The first one was the default group consisting of images with minimal artefacts. The second group consisted of images with visible out-of-focus blur. The third group covered images with a variety of imperfections stemming from artefacts from the imprinting or imaging setup. To prevent data leakage^84^ and ensure proper model evaluation, we divided the dataset into a training- (60%: 150 images), validation- (20%: 50 images) and a hold-out test-set (20%: 50 images) using a stratified split. Additional summary dataset information can be found in Supplementary Table S3. For the evaluation of stomatal density in NILs and *ZmAbh* mutants, we used separate datasets where only stomatal counts (no bounding boxes) were provided. During training, images were resized to a width of 800 pixels while maintaining their aspect ratio to match the pre-trained features. Random horizontal and vertical augmentations were applied, and the dataset was normalized using the mean and standard deviation of the training set.

We optimized key hyperparameters, including learning rate, batch size, and the number of RoI heads (region proposals from the RPN), using grid search. The optimal model was selected based on mAP50 (details in the "Evaluation metrics" section). Final training was performed using pre-trained ImageNet weights. Models were trained for at least 10,000 steps, except for one batch size, which required 15,000 steps. To save computing resources, the maximum step size was reduced from 15,000 to 10,000 where possible.

All models were implemented using Python 3.8^85^, PyTorch^86^, detectron2^87^ and were trained and tested on a Ubuntu 22.04 LTS machine with 64 Intel CPU cores, 512 GB of memory, and one NVIDIA RTX A6000 graphics card. The code and pre-trained models are publicly available under: https://github.com/grimmlab/StomaDet.

### Evaluation metrics

We used the mean Average Precision (mAP)^88^-a metric commonly used in object detection-to evaluate our models and to select the best performing one. This metric compares the predicted bounding boxes to the ground truth and is the mean of the Average Precision (AP) values over all classes in the dataset. The AP is calculated using the area under the precision-recall curve. There are different ways on how to interpolate and calculate the AP values—here we follow the method outlined in the Microsoft COCO dataset^89^ and use the IoU threshold of 0.5 (mAP50) as our main metric.

### Data evaluation

We validated model performance through two experiments. First, to evaluate detection capabilities on unseen data, we calculated the mean Average Precision (mAP) on the hold-out test set. Optimizing over 230 hyperparameter sets (top 10 shown in Supplementary Table S4), our model consistently achieved mAP values close to 99%, indicating precise stomata detection.

### Final model performance

Using the optimal hyperparameter set, we re-trained our final model on the combined training and validation sets, starting from pre-trained ImageNet weights. The final model achieved an average precision of 98.96% on the never used hold-out test set, demonstrating strong generalization. Only a few predictions were inaccurate (examples shown in Supplementary Fig. S6). Key challenges included severely blurred images (Supplementary Fig. S6 A-D), round artefacts like bubbles, and partially visible stomata at image borders. Notably, the model successfully detected stomata with approximately 50% visibility at the borders (Supplementary Fig. S6 E–H).

### Stomata counting performance

In the second experiment, we evaluated the model’s stomata counting performance using additional post-processing for improved precision. We applied a confidence threshold of > 50% and an area threshold requiring at least 50% stomatal visibility. Detection areas were approximated using bounding box dimensions, retaining only predictions larger than half the mean stomatal area. As the mean area varied by genotype and other factors, this calculation was performed per image to eliminate false positives.

To ensure robust evaluation, we analysed 1,485 microscopy images: 855 from NILs and 630 from *ZmAbh* mutants (Supplementary Table S3). As a result, the predictions of our model were highly correlated (Pearson correlation coefficient of 0.996) with manual scoring (Supplementary Fig S7).

### Statistical analyses

Statistical analyses were conducted in R Studio (v.4.3.3, R Core Team, 2024)^90^. For significance testing, multiple pairwise comparisons among genotypes were conducted using Student’s t-tests with Benjamini-Hochberg (BH) correction to control for multiple testing. For the expression data, a Tukey’s HSD test was conducted. In the experiment where both genotype and treatment (Control vs High temperature) were factors, a two-way ANOVA was performed to assess the effects of genotype, treatment and their interaction for each trait (g_s_, A and iWUE), followed by multiple pairwise comparisons among genotypes using Student’s t-tests with BH correction within treatments. ANOVA derived P-values are listed in Supplementary Table S1. The multcompView^91^ package was utilized to generate compact letter displays.

## Supplementary Information

Supplementary Information.

## Data Availability

The generated and labelled dataset and the code and pre-trained models used for the development of the deep learning-based stomata detection model are publicly available on Mendeley data https://www.doi.org/10.17632/2bfcpc6bpj.1 and on GitHub: https://github.com/grimmlab/StomaDet.
