# Supplementary material for: The combined effect of decreased stomatal density and aperture increases water use efficiency in maize
Source: Sci Rep. 2025 Apr 21;15:13804. doi: 10.1038/s41598-025-94833-1 (PMC12012185; doi:10.1038/s41598-025-94833-1)
Supplement: Supplementary file 1 — Supplementary Information. [file 41598_2025_94833_MOESM1_ESM.pdf]

The combined effect of decreased stomatal density and aperture increases water use efficiency in maize

**Larissa Barl [1,5], Betina Debastiani Benato [1,5], Nikita Genze [2,3], Dominik G. Grimm [2,3], Michael Gigl [4], Corinna Dawid [4], Chris-Carolin Schön [1], Viktoriya Avramova [*,1]**

# Supplementary Figures and Tables

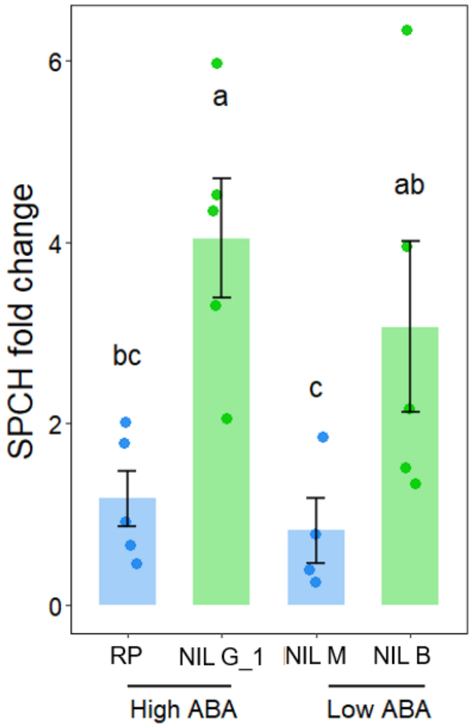


**Supplementary Figure S1: Relative expression levels of stomatal lineage transcription factor SPEECHLESS (SPCH) in the leaf base of near-isogenic lines (NILs) with different ABA levels.** Fold changes in gene expression are shown for SPCH in NILs (NIL G_1, NIL M, and NIL B) compared to the recurrent parent (RP). Bars represent the mean fold change, with error bars indicating standard error (n=5 biological replicates). Individual data points represent individual biological replicates averaged from three technical replicates. Light blue and green bars indicate genotypes with low or high stomatal density, respectively. Different letters denote statistically significant differences between groups based on a two-way ANOVA with Tukey’s HSD test ($P < 0.05$).

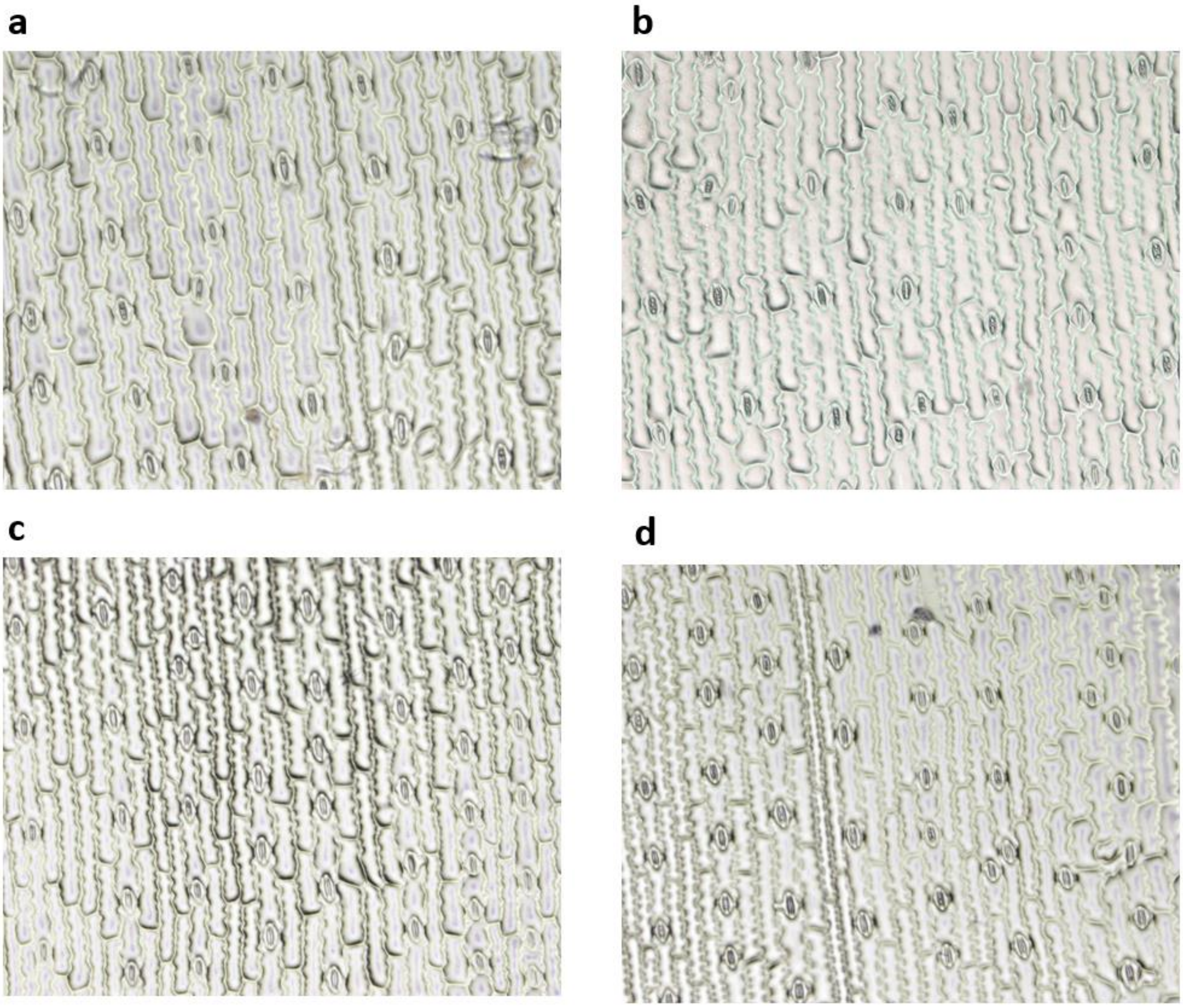

**Supplementary Figure S2: Stomatal guard cell formation of the three NILs and their recurrent parent.** Stomatal complex morphology is not affected by the introgressions present in lines with lower stomatal density RP (**a**) and NIL M (**b**) compared to lines with higher stomatal density NIL G_1 (**c**) and NIL B (**d**).

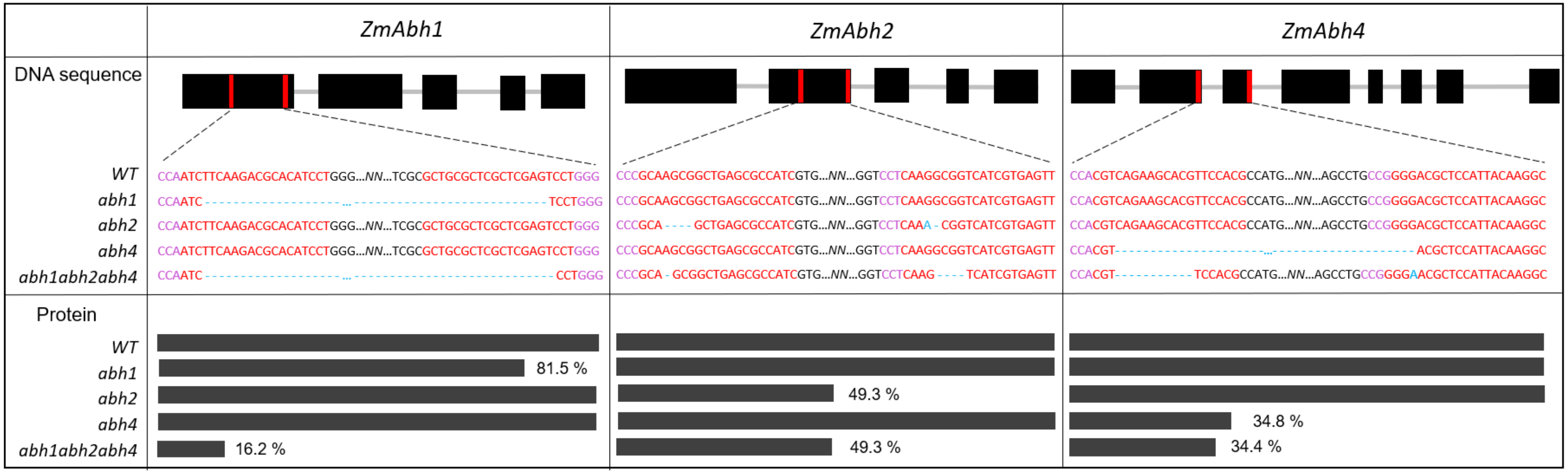


**Supplementary Figure S3: *ZmAbh* alleles of CRISPR/Cas9 mutants.** DNA sequences are given according to the B73 reference sequence (Zm-B73-REFERENCE-GRAMENE-4.0)[1]. In the DNA sequence, the exons are depicted in black, introns in light grey and the guide RNA (gRNA) complementary sequences in red. The corresponding amino acid sequence is depicted below in dark grey. Percentages indicate the remaining residues of the amino acid sequences in mutant allels. Each gene was targeted by two single gRNAs. The complements of PAM sequences are written in purple letters, the sequences matching the gRNA sequence are written in red and the mutations are highlighted in blue. The single *abh1* mutant, *abh1.27*, and the *ZmAbh1* allele in the *abh1abh2abh4* triple mutant show a deletion of 261 bp and 262 bp in the coding sequence, respectively. The 262 bp deletion causes a frameshift and an premature stop codon. The single *abh2* mutant, *abh2.21*, shows a deletion of 4 bp, a downstream G to A conversion and a downstream 1bp deletion in the coding sequence, while the *ZmAbh2* allele in the *abh1abh2abh4* triple mutant has a deletion of 1 bp and a downstream 4 bp deletion in the coding sequence, both causing a frameshift and a premature stop codon. The single *abh4.41* mutant shows a deletion of 157 bp in the coding sequence, while the *ZmAbh4* allele in the *abh1abh2abh4* triple mutant has a deletion of 11 bp and a downstream insertion of a single A in the coding sequence. The 11 bp deletion also causes a frameshift and a premature stop codon.

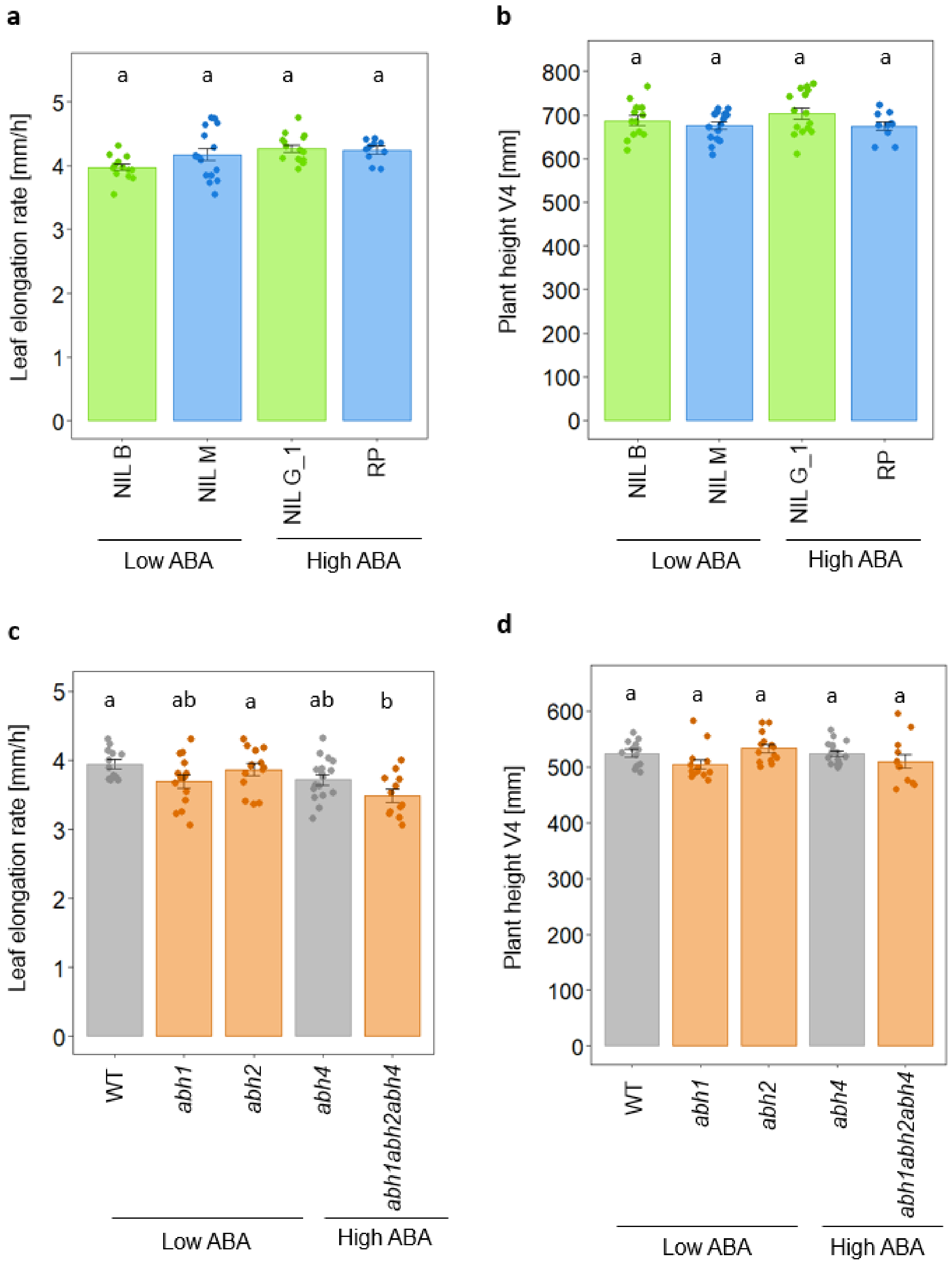


**Supplementary Figure S4: Plant growth.** Leaf elongation rate of leaf 7 of near-isogenic lines (NILs; **a**; n=10-16 plants) and null *ZmAbh* mutants and their wild type (WT; **c**; n=12-17 plants) was calculated during the first three days of its emergence. Plant height of NILs (**b**; n=10-16 plants) and null *ZmAbh* mutants and their WT (**d**; n=12-17 plants) was measured at developmental stage V4. For the NILs, light blue and green bars indicate genotypes with low or high stomatal density, respectively. For the WT and mutant lines, orange and grey bars indicate genotypes with low or high stomatal density, respectively. Bar charts show means ± SE. Different letters indicate significant differences in pairwise comparisons with Benjamini-Hochberg correction ($P < 0.05$).

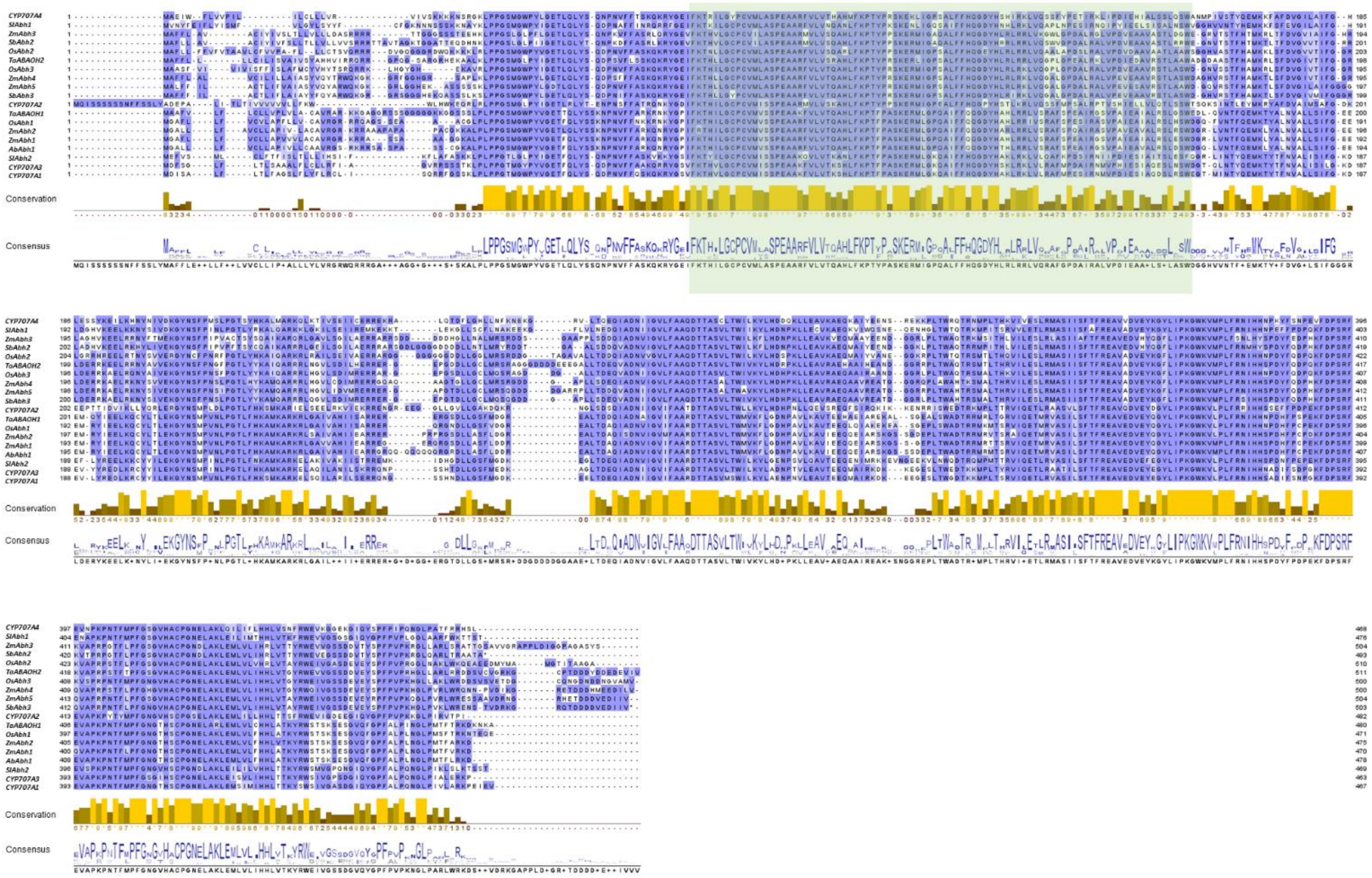

**Supplementary Figure S5: Alignment of the amino acid sequence of all members of the ABA 8'- hydroxylase gene family in maize, sorghum, rice, wheat, tomato and arabidopsis showing the conservation and consensus among the sequences.** The green box marks the deletion in the *abh1* mutant. The yellow bars below the sequences indicate conserved residues. The alignment was generated with Jalview (version 2.11.4.1) using sequences from NCBI (https://www.ncbi.nlm.nih.gov).

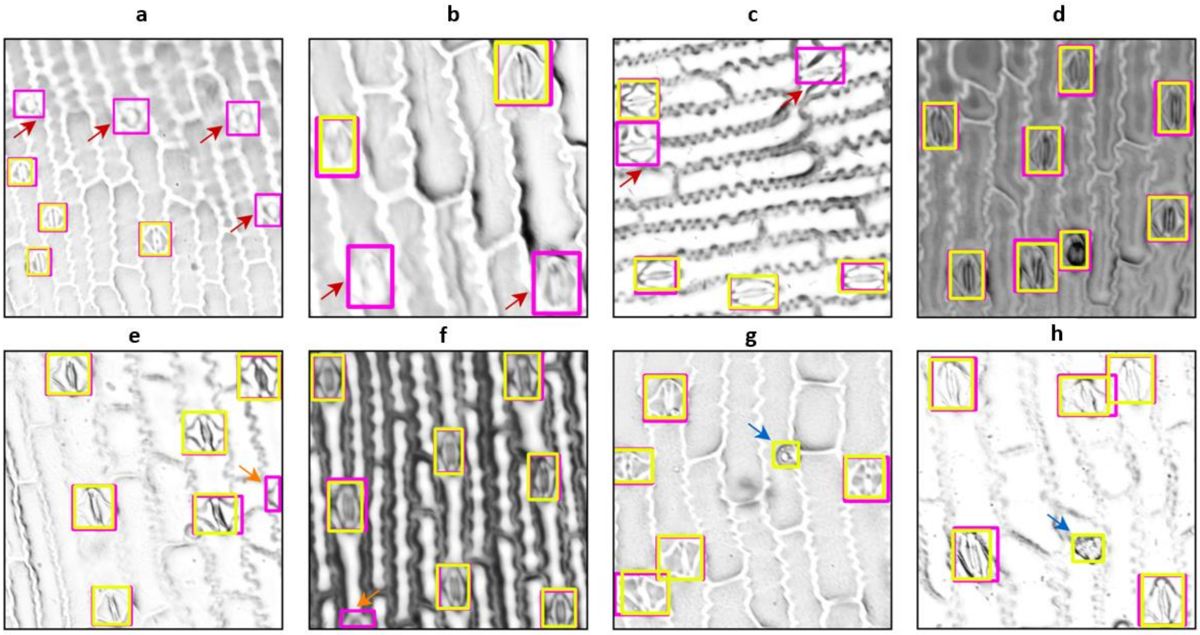


**Supplementary Figure S6: Example predictions based on the hold-out test set.** The ground truth is indicated by pink boxes, while the predictions are shown in yellow. (**a-d**) Missed detections due to severe out-of-focus blur (red arrows), while detecting stomata with moderate amount of blur (predictions bounding boxes overlap with the ground truth). (**e-f**) Missed detections due to partly visible stomata at the image edges (orange arrows). (**g-h**) Falsely detected as stomata artifacts - mostly small bubbles - in the images (blue arrows).

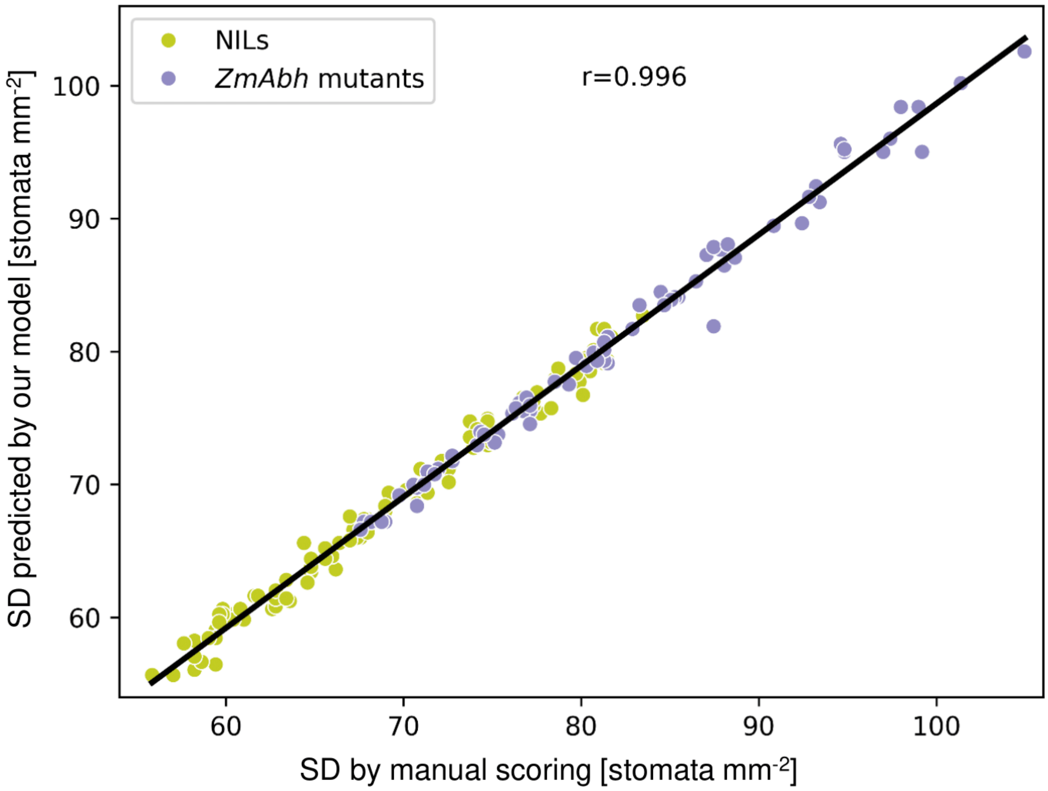


**Supplementary Figure S7: Correlation between stomata density (SD) scored manually and predicted by our deep learning-based stomata detector.** The predictions are highly correlated compared to manual scoring (Pearson correlation r = 0.996). For the NILs (green), there were 855 samples analyzed, and for the *ZmAbh* mutants (purple) 630 samples. The plotted values correspond to the mean SD of 9 pictures per sample.

**Supplementary Table S1: Two-way ANOVA results for the gas exchange traits stomatal conductance ($g_s$), $CO_2$ assimilation rate (A), and intrinsic water use efficiency (iWUE) in NILs and *ZmAbh* mutants**. P-values are provided for genotype, treatment (Control vs. High temperature), and their interaction for each trait.

| Trait | Genetic Material | P (Genotype) | P (Treatment) | P (Genotype:Treatment) |
|---|---|---|---|---|
| $g_s$ | NILs | 6.6e-10 *** | 1.47e-07 *** | 0.148 |
| | *ZmAbh* mutants | 9.46e-06 *** | 0.0007 *** | 0.4855 |
| **A** | NILs | 0.0879 . | 0.7149 | 0.1993 |
| | *ZmAbh* mutants | 0.587 | 0.0002 *** | 0.5058 |
| **iWUE** | NILs | 1.31e-09 *** | 1.7e-09 *** | 0.0835 . |
| | *ZmAbh* mutants | 3.07e-09 *** | 0.0845 . | 0.7315 |

**Supplementary Table S*2*: Regions with genomic introgressions from a donor parent in the background of a recurrent parent in three near isogenic lines (NILs).** Recombination breakpoints and introgression lengths were determined by genotyping with the 600 k Axiom™ Maize Genotyping Array[2]. Physical coordinates are given according to the B73 reference sequence (Zm-B73-REFERENCE-GRAMENE-4.0)[1].

| | **Introgression** | | |
|---|---|---|---|
| **Genotype** | **Start (bp)** | **End (bp)** | **Length (bp)** |
| NIL B | 110,755,114 | 166,111,057 | 55,355,943 |
| NIL G_1 Introgression 1 | 110,755,114 | 129,917,254 | 19,162,140 |
| NIL G_1 Introgression 2 | 129,919,739 | 133,999,580 | 4,079,841 |
| NIL M | 129,798,239 | 129,994,822 | 196,708 |

**Supplementary Table S3: Summary of the annotated dataset used to train and evaluate the stomata detector**

| | **Images** | **Stomata** |
|---|---|---|
| **Training** | 150 | 6546 |
| **Validation** | 50 | 2202 |
| **Test** | 50 | 2045 |
| **NILs** | 855 | 33149 |
| ***ZmAbh* mutants** | 630 | 28917 |

**Supplementary Table S4: Top-10 hyperparameter sets found.** The complete results are stored in our github repository (https://github.com/grimmlab/StomaDet).

| Iteration step | Learning rate | Batch size | ROI heads | AP (IoU=50) in percent |
|---|---|---|---|---|
| 9800 | 0.00200 | 1 | 256 | 98.9903 |
| 6200 | 0.00446 | 1 | 128 | 98.9898 |
| 5400 | 0.00255 | 1 | 256 | 98.9895 |
| 2400 | 0.00367 | 1 | 128 | 98.9894 |
| 5200 | 0.00198 | 1 | 512 | 98.9877 |
| 5000 | 0.00118 | 2 | 128 | 98.9876 |
| 6800 | 0.00367 | 1 | 256 | 98.9876 |
| 9800 | 0.00270 | 1 | 512 | 98.9872 |
| 3200 | 0.00367 | 2 | 256 | 98.9872 |
| 2600 | 0.00367 | 2 | 128 | 98.9869 |

## References:


1 Jiao, Y. *et al.* Improved maize reference genome with single-molecule technologies. *Nature* **546**, 524-527 (2017).

2 Unterseer, S. *et al.* A powerful tool for genome analysis in maize: development and evaluation of the high density 600 k SNP genotyping array. *BMC Genomics* **15**, 823 (2014).
